# Batch effect correction for genome-wide methylation data with Illumina Infinium platform

**DOI:** 10.1186/1755-8794-4-84

**Published:** 2011-12-16

**Authors:** Zhifu Sun, High Seng Chai, Yanhong Wu, Wendy M White, Krishna V Donkena, Christopher J Klein, Vesna D Garovic, Terry M Therneau, Jean-Pierre A Kocher

**Affiliations:** 1 Division of Biomedical Statistics and Informatics, Department of Health Sciences Research, Mayo Clinic College of Medicine, 200 First Street, Rochester, MN 55905, USA; 2 Genomic Shared Resources, Mayo Clinic College of Medicine, 200 First Street, Rochester, MN 55905, USA; 3 Division of Maternal Fetal Medicine, Department of Obstetrics & Gynecology, Mayo Clinic College of Medicine, 200 First Street, Rochester, MN 55905, USA; 4 Department of Urology, Mayo Clinic College of Medicine, 200 First Street, Rochester, MN 55905, USA; 5 Department of Neurology, Mayo Clinic College of Medicine, 200 First Street, Rochester, MN 55905, USA; 6 Division of Nephrology and Hypertension, Department of Internal Medicine, Mayo Clinic College of Medicine, 200 First Street, Rochester, MN 55905, USA

## Abstract

**Background:**

Genome-wide methylation profiling has led to more comprehensive insights into gene regulation mechanisms and potential therapeutic targets. Illumina Human Methylation BeadChip is one of the most commonly used genome-wide methylation platforms. Similar to other microarray experiments, methylation data is susceptible to various technical artifacts, particularly batch effects. To date, little attention has been given to issues related to normalization and batch effect correction for this kind of data.

**Methods:**

We evaluated three common normalization approaches and investigated their performance in batch effect removal using three datasets with different degrees of batch effects generated from HumanMethylation27 platform: quantile normalization at average β value (QNβ); two step quantile normalization at probe signals implemented in "lumi" package of R (lumi); and quantile normalization of A and B signal separately (ABnorm). Subsequent Empirical Bayes (EB) batch adjustment was also evaluated.

**Results:**

Each normalization could remove a portion of batch effects and their effectiveness differed depending on the severity of batch effects in a dataset. For the dataset with minor batch effects (Dataset 1), normalization alone appeared adequate and "lumi" showed the best performance. However, all methods left substantial batch effects intact in the datasets with obvious batch effects and further correction was necessary. Without any correction, 50 and 66 percent of CpGs were associated with batch effects in Dataset 2 and 3, respectively. After QNβ, lumi or ABnorm, the number of CpGs associated with batch effects were reduced to 24, 32, and 26 percent for Dataset 2; and 37, 46, and 35 percent for Dataset 3, respectively. Additional EB correction effectively removed such remaining non-biological effects. More importantly, the two-step procedure almost tripled the numbers of CpGs associated with the outcome of interest for the two datasets.

**Conclusion:**

Genome-wide methylation data from Infinium Methylation BeadChip can be susceptible to batch effects with profound impacts on downstream analyses and conclusions. Normalization can reduce part but not all batch effects. EB correction along with normalization is recommended for effective batch effect removal.

## Background

DNA methylation, as one of the major epigenetic mechanisms of DNA modification, plays a significant role in gene expression regulation, organism development, X chromosome inactivation, and genetic imprinting in vertebrates [1]. Changes in methylation patterns and levels have been shown to be associated with various diseases such as cancers [2-6] and genetic disorders [7,8]. Coupled with gene expression microarrays, genome-wide methylation profiling provides the ability to better understand the delicate and complex mechanisms of gene expression regulation and to search for therapeutic targets [3,4,9].

The Illumina HumanMethylation27 BeadChip is one of the most commonly used genome-wide methylation profiling platforms in the literature. Thousands of TCGA (The Cancer Genome Atlas) samples have been assayed using this platform. This bead chip measures the methylation status of over 27,000 CpG sites in the genome at single nucleotide resolution [10]. At each CpG site, average β is obtained to represent the methylation level through two types of probe targeting methylated and unmethylated cytosines (C for methylated and T for unmethylated) respectively after bisulfite treatment of a sample. Because the average β is the measurement within a sample and the two probe signals at a particular CpG are reported by the same dye, it is generally believed normalization is not necessary. However, when a study has a number of samples, they need to be allocated into different chips or processed in different times. Like other high throughput genomic data [11], this inevitable logistics can cause various batch effects as reported in the literature [2,9,12] and from our experience with multiple datasets including methylation data from TCGA. Batch effects are the technical artifacts that are not associated with the underlying biology, but rather to unrelated factors, such as laboratory conditions, experiment time, reagent lots and/or laboratory personnel differences. The impact of these factors sometimes can be so profound that, without appropriate correction, they may lead to inaccurate conclusions or a significant reduction in the power for true biological signal detection. Since batch effects can affect different probes in different ways, they often can not be corrected through routine normalization methods and special techniques are needed [2,11]. Several batch effect correction methods have been developed for gene expression microarray [13-17]; however, the appropriateness of these methods for the methylation data has not been evaluated. What makes more complex is the debate whether or which normalization should be conducted before batch effect correction. The practice of normalization in the literature is highly variable, with some studies without mentioning any normalization [3,4,6,7,18], and others with quantile or lowess normalization on average β [2,9,12,19] or at probe signal intensities [8]. In the recent release of R package "lumi" [20], more complex signal channel adjustment and then normalization on pooled two signals are proposed http://www.bioconductor.org/packages/release/bioc/html/lumi.html. However, the appropriateness and performance of these normalizations are little known.

In this study, we presented three Illumina Human Methylation27 datasets with different degrees and patterns of batch effects, and investigated the application of quantile normalization on average β, signal intensities as implemented in R package "lumi", and separate methylated and unmethylated signals. We demonstrated each of these normalizations reduced technical artifacts yet with some differences. None of these methods were able to remove refractory batch effects and specialized batch removal was required. The Empirical Bayes (EB) [17] batch correction method was shown an effective way to correct batch effects along with normalization.

## Methods

### Illumina methylation platform

Human Methylation27 BeadChip is an allele specific assay with 27,578 CpG loci covering more than 14,000 genes [10]. Each chip (or array) can accommodate up to 12 samples. The assay detects the methylation status of individual CpG by typing bisulfite-converted DNA. Methylation protects cytosine (C) from conversion, whereas unmethylated C is converted to T during the bisulfite treatment. A pair of bead-bound probes is used to quantify the amount of T or C through hybridization. Signal intensities from the two bead types are obtained from BeadArray Reader. The methylation status of a CpG site is determined from the average β-value (bounded between 0 and 1), which is the ratio of the fluorescent signal of the methylated probe to the total intensity by both probes.

### Datasets

The three datasets evaluated in this study were selected from three studies conducted previously at Mayo Clinic and each was approved by the Mayo Clinic Institutional Review Board (IRB). All were generated using the Human Methylation27 bead chip in the Genotyping Shared Resources (GSR) at Mayo Clinic.

#### Dataset 1

This dataset consisted of 84 blood samples from pregnant and non-pregnant women (either never pregnant or over different time points during pregnancy), among which 9 were technically replicated for quality control (a total of 93 arrays). The study subjects between cases and controls were age (25+/-1) and body mass index (26+/-1) matched and the samples were randomly allocated to 8 bead chips and processed at the same time. The samples on one chip tended to form a separate cluster from other samples in principal components analysis but no such cluster was seen in unsupervised clustering, suggesting some minor batch effects from this chip. Four of the 9 technical replicates were located on this chip. This dataset was used to assess the performance of normalizations and EB batch correction by comparing the 9 technical replicate pairs. For the two chips we further investigated, Chip22 had 7 samples from pregnant and 5 from non-pregnant women; Chip26 had 5 samples from pregnant and 7 from non-pregnant women.

#### Dataset 2

Twenty four blood samples, 14 from cases with a neurological disorder and 10 from controls, were randomly allocated to two chips (6 cases and 6 controls on Chip11 and 8 cases and 4 controls on Chip12). The case and controls were age matched (+/- 5 years olds). All samples were processed in the same way except that the two chips were hybridized one day apart. The study included both male and female. To reduce the effects of differential methylation between male and female on sex chromosomes, CpGs on chromosome X and Y were excluded for all analyses for this dataset, which left 26,486 CpGs.

#### Dataset 3

Two chips of 24 samples, 18 from prostate cancers and 6 from normal prostates (adjacent normal from some cancer patients), were selected from a study where two big batches of chips were purchased and hybridized roughly 6 months apart. One chip from one batch contained 8 tumors and 4 normal tissues and the other from another batch had 10 tumors and 2 normal samples.

### Data pre-processing and initial quality assessment

Illumina's BeadStudio (version 3.1.3) with methylation module (version 3.2.0), or GenomeStudio (version 2010.1) with methylation module (version 1.6.1), was used to process the raw image data generated by BeadArray Reader. Initial quality assessment of assay performance was conducted using the "Control Dashboard" in the software package, which includes graphical inspection of 8 types of embedded control probes (staining, hybridization, target removal, extension, bisulfite conversion, G/T mismatch, negative control, and non-polymorphic controls). Overall sample quality was determined through total number of detected CpGs, average detection p-value across all CpG sites, and the distribution of average β for all CpGs. All samples in this study passed these basic quality assessments.

### Batch effect assessment

After the initial QC, we assessed overall methylation profile for batch effects. In this study, a batch was defined as a set of 12 samples from the same chip (note that batch effects may not be always at chip level; based on how samples are handled, they can occur by a plate of 96 samples or a set of chips). We used several metrics to evaluate batch effects for each dataset: (a) the distribution of average β values for all samples in one batch contrasting with another through a box and density plot, (b) unsupervised hierarchical clustering of all CpGs (27,578 for Dataset 1 and 3; and 26,486 for Dataset 2) using 1 minus Pearson correlation distance matrix and average linkage, (c) a principal components analysis (PCA) plot of the first few (2-3) principal components using all CpGs, (d) the associations of first 10 principal components with the batch variable by Wilcoxon test at a p value < 0.01, and (e) the proportion of CpGs significantly associated with batches by applying analysis of variance (ANOVA) test on each CpG and then counting those with a p value < 0.01 among all 27,578 CpGs (26,468 for Dataset 2 after excluding CpGs on Chr X and Y).

### Normalization

We first evaluated three quantile normalization strategies for Dataset 1 with 93 samples where 9 pairs of technical replicates were used as a measure of performance: 1) directly on average β (QNβ). Similar to gene expression microarray, this normalization makes the distribution of CpG β values for each sample in the dataset the same [21]; 2) two color (red and green) channel signal adjustment first and then quantile normalization on pooled signals of both and recalculation of average β as implemented in "lumi" package of R (lumi) [20]; 3) separate quantile normalization on methylated (B signal) and unmethylated (A signal) signals and then re-calculation of average β (ABnorm). For both lumi and ABnorm, average β values were recalculated from normalized signal data using the same formula as implemented in the Illumina Beadstudio or GenomeStudio (average β = signal B/(signal A + signal B + 100)). The performance of each normalization was evaluated by comparing the errors of all CpGs between 9 technical replicate pairs, which were obtained by calculating the differences for each of CpGs between two replicates and they should be zeros theoretically. Because no technology can achieve that precision, the criteria were to examine whether the mean of the errors between two replicates shifted from zero and how large the deviations were. We used two measures for each replicate pair: 1) the mean of the errors; 2) the average absolute deviation of the errors from zero, which is the sum of absolute values of the errors divided by the number of total CpGs. The three normalization methods were then applied to Dataset 2 and 3 with more obvious batch effects.

### Batch correction using EB algorithm

Quantile normalization adjusts measurements at a global level. In other words, it is not designed to remove artifacts that only affect a subset of probes or genes [11]. For Dataset 2 and 3, all normalization methods failed to correct a large proportion of batch effects and we accounted for this subsequently by applying the EB correction method [17]. We selected this method as it is the most flexible with the best performance among the commonly used batch correction methods in gene expression microarray [22]. The outcome of interest, i.e, the case control status for Dataset 2, and tumor and normal status for Dataset 3, was incorporated into the batch correction model so that the biological information was taken into account.

### Performance evaluation of normalization and batch correction

In addition to the evaluation of technical replicates for Dataset 1, we used the same evaluation metrics as described in the section "Batch effect assessment" to determine the adequacy of normalization and EB batch correction after normalization and/or EB correction was conducted on each dataset. To measure the improvement of biological signal detection, we applied ANOVA test for differentially methylated CpGs between case and control of Dataset 2 and prostate cancer and normal prostate for Dataset 3 before and after normalization and EB correction and recorded the proportion of CpGs associated with these outcome variables (p value < 0.01 among all analyzed CpGs).

## Results

### Patterns and severity of batch effects

Obvious batch effects are often indicated from basic QC plots such as a density or box plot which shows a different distribution of average β values in one batch from another regardless of sample types. In PCA plot or unsupervised clustering, samples from the same batch form a distinct cluster.

**Figure 1:**
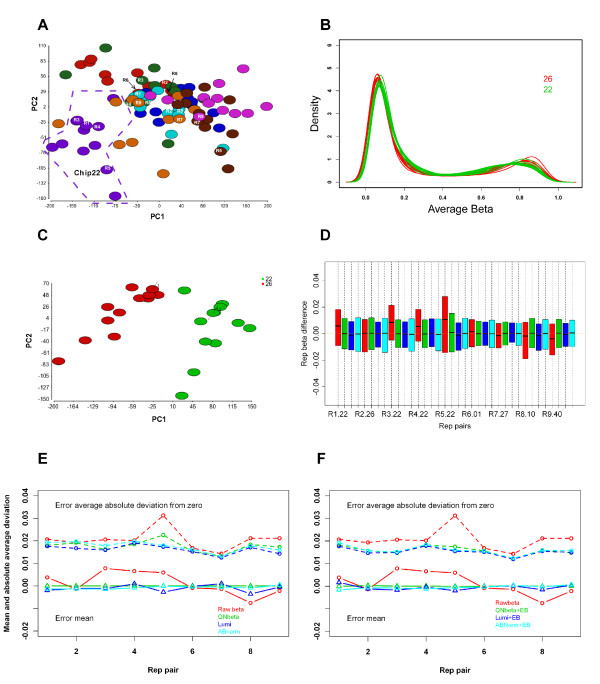
**Dataset 1 before and after normalization and batch effect correction**. A: PCA plot for all 93 samples using all 27,578 CpGs. Different colors are for different batches. Nine pairs of technical replicates are marked as R1 to R9. The samples on Chip12 (circled with dash line) tend to separate from other samples. B. Density plot of samples from Chip12 and Chip26 shows minor distribution biases between the two chips. C: PCA plot of the 24 samples from Chip22 and 26 using all 27,578 CpGs. Two samples with an across bar are technical replicates. D: Box plot of pair-wise CpG errors between 9 pairs of technical replicates for unnormalized average β (red), QNβ (green), lumi (blue), and ABnorm (cyan). The unnormalized data has wider interquartile ranges and shifted medians from zero line. All normalized data have condensed interquartile ranges with medians adjusted close to zero line. E: Error means (lower pane) and average absolute deviations (upper panel) of 9 pairs of technical replicates before (red) and after three normalizations. Unnormalized data has the largest average absolute deviation for each of replicate pairs and shifted mean for most of the pairs. All normalized data show reduced average absolute deviations. F: Error means (lower pane) and average absolute deviations (upper panel) of 9 pairs of technical replicates before and after three normalizations plus EB correction. The normalized and EB correction data have almost identical error means and average absolute deviations compared to normalized data alone.

The batch effects in Dataset 1 were very subtle. In the PCA plot, the samples on Chip22 tended to separate from other samples (Figure 1A); however, this was not visible in unsupervised clustering. For better visualization, we extracted the data from this chip and another chip (Chip26). As shown in Figure 1B, the density plot of average β values showed a slight shift and separation between the samples from the two chips. The batch effects were more visible in the PCA plot for the two chips (Figure 1C). Note that two technical replicates (with across bars) were apart in the first principal component and clustered more tightly to the samples on their respective chip.

**Figure 2:**
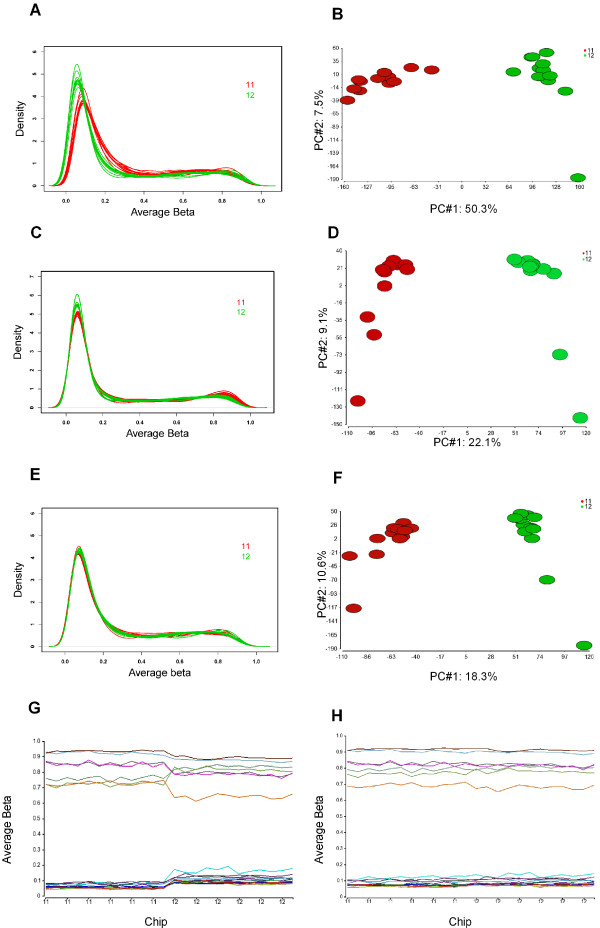
**Dataset 2 before and after normalization and batch correction**. A: The density plot of average β values for two chips of samples. All samples on Chip12 shift to the left, more CpGs with lower methylation values. B: PCA plot (first two components) for the 24 samples using 26,486 CpGs after excluding CpGs in sex chromosomes; samples on each chip cluster closely with the first component explaining 50.3% of variance. C: Density plot of lumi normalized data. The distribution bias has been greatly reduced but a significant portion remains. D: PCA plot of lumi normalized data still shows clear sample separation by batches using 26,486 CpGs after excluding CpGs in sex chromosomes. E: Density plot of average β after ABnorm. The distribution bias has been successfully removed. F: PCA plot of ABnorm data shows the clear remaining batch effects using 26,486 CpGs. G: The profiles of selected 20 CpGs that are associated with the batch effects after normalization. X-axis-samples ordered by Chip (11 or 12). Y-axis-methylation average β. Each line represents one CpG across samples. These CpGs are either all higher or lower on one chip than another. H: The profiles of the same 20 CpGs as G after normalization and EB correction. The systematic differences between the two chips have been removed.

For Dataset 2, the samples on Chip12 had lower average β values than those in Chip11 and shifted to the left in the density plot (Figure 2A). In both unsupervised clustering and PCA plot, samples from Chip11 and 12 were clearly separated into two clusters (Figure 2B, PCA was shown only). Note that the case and control samples were in the similar ages and were scattered in two chips; the separation was clearly due to experimental artifacts. The M-A plot showed an "intensity" dependent bias of average β values between the two chips (Additional file 1A).

**Figure 3:**
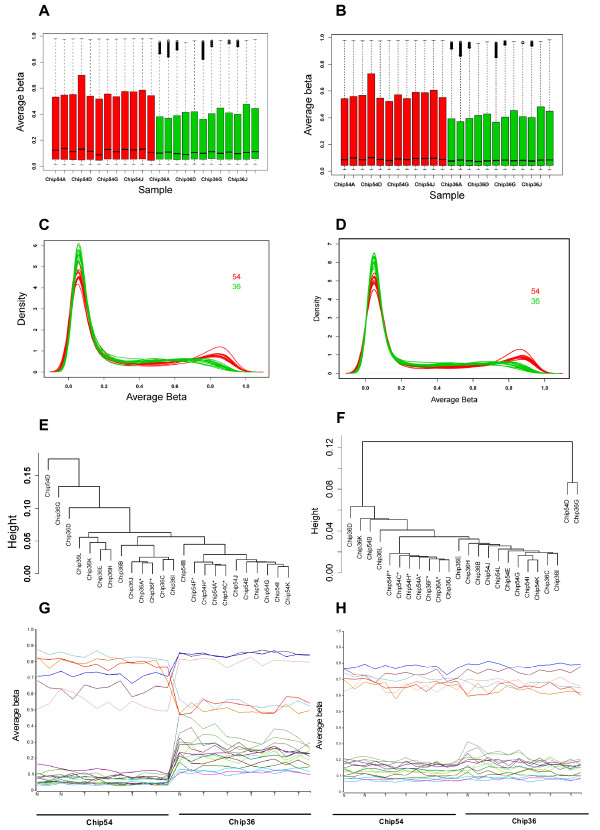
**Dataset 3 before and after normalization and batch effect correction**. A: Box plot of raw average β for two chips of 24 samples. The medians between the two chips are similar, but the 3rd quartile values of Chip36 are much lower than Chip54. B: Box plot for the two chips of 24 samples after lumi normalization. C: The density plot of average β for 24 samples before normalization colored by batches. The distribution differs obviously between the chips. D: The density plot of "lumi" normalized average β for 24 samples shows a large portion of batch effects not corrected. E: unsupervised clustering using all 27,578 CpGs before normalization and EB correction shows the clear separation of samples by chips (Chip54 or Chip36); samples from the same tissue type tend to cluster within the same batch (* for normal prostates and others for tumors). F: unsupervised clustering after normalization and EB correction using all 27,578 CpGs shows the separation between batches is removed; samples from the same tissue type cluster closely (* for normal prostates and others for tumors). G: Methylation profiles of selected 20 CpGs that are significantly associated with the batch effects after normalization, showing the dramatic differences between the two chips. The letter "T" and "N" on x-axis represent tumor and normal sample. H: The methylation profiles of the same 20 CpGs as G after the additional EB correction. The systematic biases are successfully removed.

Dataset 3 presented a different distribution from Dataset 2 where the medians of average β values between the two chips were similar, yet all samples on Chip36 (green color) had much smaller upper quartiles (Figure 3A, C). As expected, unsupervised hierarchical clustering showed grouping of samples from the same chip regardless they were from prostate cancer or normal tissues (Figure 3E). Again, an obvious systematic bias was seen from the M-A plot between the two chips (Additional file 1B).

As quantitative measures of batch effects, we counted the percentage of CpGs that were associated with the batch effects for Dataset 2 and 3. As shown in Table 1 column "Raw β", the two datasets had 66% and 50% of CpGs associated with the batch variable at p value less than 0.01, respectively. The first principal component of both was significantly associated with the batches from Wilcoxon test for the first 10 components examined.

**Table 1:** Statistical measures of batch effects and performance evaluation of normalization and batch correction

Dataset	Statistical measure	Raw β	QNβ	Lumi	ABnorm	QNβ+ EB	Lumi+ EB	ABnorm+ EB
	Number (%) of CpGs associated with batch at p < 0.01	17,458 (66)	6,466 (24.4)	8,478 (32)	6,926 (26)	12	25	23
**2**	PCs associated with batch(% variance explained)*	1 (51.6)	1 (17.9)	1 (22.1)	1 (18.9)	None	None	None
	Number (%) of differentially methylated CpGs between case and control at p < 0.01	345 (1.3)	759 (2.9)	714 (2.7)	763 (2.9)	1,155 (4.2)	1,146 (4.3)	1,229 (4.6)

	Number (%) of CpGs associated with batch at p < 0.01	13,881 (50.0)	10,300 (37.3)	12,668 (46)	9,694 (35.2)	2	6	8
**3**	PCs associated with batch (% variance explained)	1 (50.4)	1 (24.8)	1 (30.6)	1 (23.8)	None	None	None
	Number (%) of differentially methylated CpGs between cancer and normal at p < 0.01	794 (2.9)	1,877 (6.8)	1,131 (4.1)	1,635 (5.9)	2,799 (10.1)	2,400 (8.7)	2,289 (8.3)

Raw β: Raw average β without any correction; QNβ: quantile normalization at average β values; lumi: two step quantile normalization at probe signals implemented in R package "lumi"; ABnorm: quantile normalization for A and B signal separately; EB: Empirical Bayes batch correction. * The principal components (PC) significantly associated with batch effects at p value < 0.01 from the top 10 evaluated by Wilcoxon test and the percentage of variance the PC explains.

### Comparison of different normalizations on Dataset 1 with technical replicates

We applied three normalization strategies to Dataset 1 with all 93 samples together. The purpose was to compare the errors (pair-wise average β value differences) between 9 replicate pairs before and after normalization. In the box plot of the errors (Figure 1D), for every replicate pair the un-normalized data (red color) had the largest inter-quartile range and its median shifted off the zero line the most. The spread was shrunken and the median was adjusted closer to zero by each of the normalization methods (green for QNβ, blue for lumi, and cyan for ABnorm) although there were slight differences among the three methods. Similarly, the error means deviated from zero the most for the un-normalized data. These were corrected by each of normalization methods, with QNβ leading to a straight line at zero (Figure 1E, green solid line, lower panel). The average absolute deviations (from zero) of these errors were also reduced through each of the three normalizations, with lumi normalization having the smallest average absolute deviation in most of replicates pairs (Figure 1E, upper blue dashed lines). However, the error means from this normalization deviated more from zero line than QNβ and ABnorm. Note that the replicates on Chip22 (one of the pair 1, 3, 4, 5), which demonstrated some batch effects, had higher deviations than the replicates on other chips and these were significantly reduced after normalizations. After each of the normalizations, the distribution bias and the cluster of Chip22 were no longer present, suggesting the minor batch effects could be corrected by simple normalization.

### Normalization not sufficient for data with obvious batch effects

Although the three normalizations performed similarly in reducing the batch effects for Dataset 1, the results for Dataset 2 and 3, which both had obvious batch effects, showed some clear differences. While QNβ adjusted each sample to identical distribution as expected, lumi normalization was not able to remove the distribution bias as shown in the box and density plots for both datasets (Figure 2C and 3B and 3D). These biases were successfully adjusted by ABnorm (Figure 2E). Each normalization removed a significant portion of technical artifacts for the two datasets, as measured by significantly reduced percentages (or numbers) of CpGs associated with the batch effects (from 66% to 24.4%, 32% and 26% for Dataset 2 and from 50% to 37.3%,46% and 35.2% for Dataset 3 using QNβ, lumi, and ABNorm normalization, respectively (Table 1). The reductions for lumi were the smallest among the three and QNβ and ABnorm were very similar.

In spite of the significant reductions, the numbers of CpGs remaining associated with batch effects were still much higher than expected. In fact, clear batch effects could be seen from PCA plot (Figure 2D for lumi and F for ABnorm, Dataset 2) or unsupervised clustering and the first principal component from PCA was significantly associated with the batch effects in both datasets regardless of normalization methods (Table 1). Noted is that the remaining batch effects after lumi normalization were larger than QNβ and ABnorm normalized data. This was further indicated by a higher proportion of CpGs associated with batch effects (32% vs. 24% and 26% for Dataset 2 and 46% vs. 37% and 35% for Dataset 3, respectively, Table 1).

### Empirical based batch correction

The results above demonstrated that batch affected CpGs were not easily corrected by global normalization methods and they had to be further corrected for reliable biological signal detection. We subsequently applied the EB algorithm to the normalized data by three normalization methods to Dataset 2 and 3. For each dataset after the step we again evaluated the batch effects and the CpGs associated with the outcome of interest. As summarized Table 1 none of the PCs were associated with the batch effects anymore for the batch corrected data; and the CpGs that were associated with the batch effects were reduced to a few (12, 25, 23 in Dataset 2 and 2, 6, 8 in Dataset 3 for QNβ, lumi and ABnorm normalized data, respectively). At the same time, the CpGs associated with the outcome of interest were dramatically increased: for Dataset 2, there were 759, 714, and 763 CpG associated with case and control status for QNβ, lumi, and ABnorm normalized data, respectively. These numbers were increased to 1,155, 1,146, 1,229, representing 52%, 61% and 61% increase. Compared to the un-normalized raw average β where only 345 CpGs were significant at p < 0.01, these were more than 3 fold increase of detected CpGs. Similarly, for Dataset 3, there were only 794 CpGs associated with prostate cancer/normal prostate for the raw data. After normalization, this number increased to 1,877, 1,131, 1,635, respectively, for QNβ, lumi and ABnorm, which further jumped to 2,799, 2400, and 2289 after EB correction. The largest leap in the normalization step was from QNβ; the changes from lumi were the most modest among the three; and ABnorm performed between the two.

As a further check of the effectiveness of EB correction, we conducted unsupervised hierarchical clustering for Dataset 3 after normalization and EB correction. The tumor and normal prostate samples in the dataset were expected to form distinct clusters as two types of tissues have very different methylation profiles [23]. This was the cases for the samples in the same batch. However, for the raw and normalized data, the normal samples were separated into two main clusters dictated by batches (Figure 3E, normal samples marked with *). After the EB correction, the normal prostate samples clustered much closer and samples from two batches were no longer segregated (Figure 3F).

To illustrate how batch affected CpGs behaved after normalization and batch effect correction in Dataset 2 and 3, we plotted the β values of the top 20 batch associated CpGs across samples ordered by batches (Figure 2G, H for Dataset 2 and Figure 3G, H for Dataset 3). For the data after normalization (Figure 2G and 3G), these CpGs were either all higher or lower consistently in one batch compared to the other (these patterns were not related to tumor or normal status and the differences between samples within a batch were very small). However, these systematic biases were adjusted to comparable levels between the two batches after the EB correction (Figure 2H and 3H)

## Discussion

In this study, we evaluated three commonly used normalization approaches in batch effect correction on Illumina Infinium methylation data. We first used the technical replicates included in Dataset 1 to assess the reduction of technical artifacts from these normalizations. Compared to raw average β, we observed each of the normalizations reduced the errors measured by average absolute deviation from zero (Figure 1D and 1E). Lumi outperformed QNβ and ABnorm for this dataset with minor batch effects. However, when a dataset had more obvious batch effects such as Dataset 2 and 3, lumi normalization became less effective, leaving a larger portion of batch effects intact. In this case, QNβ and ABnorm appeared more effective. ABnorm was less aggressive than QNβ as it performed at signal intensities.

Unlike gene expression microarray where normalization is routine practice, normalization for Infinium methylation data has been controversial. One reason is that for a particular CpG the same dye is used for both methylated and unmethylated probes and some believe no dye bias adjustment is needed [24]. Additionally, methylation measure is a ratio between methylated and unmethylated signal within a sample and it is presumed less affected by technical issues. The concern as to the appropriateness of normalization assumptions for methylation data is also raised [25]. However, growing evidence suggests that technical issues can affect methylated and unmethylated signals differently and the magnitude of these effects is too large to be ignored. For the datasets (Dataset 2 and 3) in this study, we not only observed the clear technical artifacts from batches, but also some variations between samples in the same batch that were not explained by underlying biology. This was further evidenced by the clear differences between technical replicates in Dataset 1. When M-A plots were investigated, these systematic biases showed an intensity dependent manner (Additional file 1). All these suggest the necessity of normalization. In evaluating methylation profiles of multiple individuals from the same or different tissues, a study showed that the majority of CpG methylation patterns were conserved [26], which implies that the quantile normalization assumption that the majority of markers in an experiment are not differentially expressed and should have similar distributions largely holds for methylation data, particularly when it is conducted in the same or similar tissue type. This was also supported from our presented data. For example, in Dataset 1, the variation of each CpG across 83 individuals was very small (standard deviation < 0.05 for over 95% of 27,578 CpGs). When differential methylation was conducted between case and control samples, only a small proportion (2.2%) of CpGs was with p value < 0.01. Even for the case of tumor and normal samples where a large difference is expected in Dataset 3, the differential CpGs accounted for < 10% of total (after normalization and batch correction). QNβ is an aggressive normalization approach yet could be very effective if the underlying assumption of β value distribution across samples hold, particularly when technical artifacts are severe. Normalization at signal intensities would correct the root cause of signal intensity bias and lead more accurate adjustment as the transformation from signal intensities to average β is a non-linear process and the sources of the bias become obscure once transformed. The differences between lumi and ABnorm are that lumi first conducts color channel adjustment (smooth quantile) and then pools methylated and unmethylated probes together for quantile normalization, assuming the pooled probes have similar distribution across samples. ABnorm assumes the distribution of A or B signals should be similar across samples. Although lumi normalization performed better for the dataset with minor batch effects such as for Dataset 1, it was the least effective to remove obvious distribution bias as seen in Dataset 2 and 3. ABnorm, on the other hand, could effectively correct the distribution biases for both datasets and its performance was between QNβ and lumi.

In spite of the clear benefit from each of the normalizations, it is worth pointing that normalization can be a double-edged sword, which not only does good but also harm when used improperly. Investigators should make sure the samples to be normalized together are "similar" enough (i.e., the same or similar type of tissue or patients in the similar characteristics) so that biological signals will not be compromised through normalization. Evaluation of the known "positive" (hypermethylated) and "negative" (no methylation) CpG methylation patterns in the data may help assess the effectiveness and potential bias from normalization [27]. For example, we applied this approach to Dataset 3 where many genes are known to be hypermethylated in prostate cancer compared to normal prostate. Eighty-five CpGs from 14 hypermethylated genes [28,29] and 358 CpGs from a list of house-keeping genes whose CpGs are presumably not methylated [30] were selected for differential testing. Normalization and normalization/EB correction significantly increased the numbers of differentially methylated positive CpGs compared to unnormalized data (up to 60% increase) yet the numbers of significant negative CpGs remained in the expected type I error rate (< expected 18 and p values were uniformly distributed, Additional file 2).

Batch effects appear a common phenomenon in high throughput genomic data as described recently [11]. The batch affected probes or markers tend to behave differently under different conditions and most global normalizations are not able to correct such irregular probes. For example, after normalization, 24-46% of total CpGs remained associated with batch effects in Dataset 2 and 3 depending on which normalization was used (Table 1), which makes further batch removal mandatory. Several batch correction methods have been developed for gene expression microarray, which include the EB approach applied in this work, distance weighted discrimination (DWD) [14], mean-centering [16], geometric ratio-based method [31], and surrogate variable analysis (SVA) [15]. The performance of these methods in gene expression microarray has been extensively evaluated [22]. The similar characteristics of batch effects in methylation to gene expression suggest that these methods can be applied to methylation array. Their usage is mainly dictated by their flexibility of handling the unique methylation data and performance in gene expression microarray. Methylation average β values are bounded from 0 to 1. Algorithms that generate data out of the bounds after batch correction are problematic. Besides the factor of study interest, methylation can be affected by other factors such as age and gender. These factors may need to be taken into consideration while batch correction is performed for accurate adjustment. Batch correction methods that can not incorporate these factors are less ideal. None of the aforementioned methods except the EB approach have this flexibility. The EB approach has been shown outperforming all other methods in terms of precision and accuracy [22] because it models both additive and multiplicative effects. What makes EB algorithm more attractive is that it corrects batch effects by borrowing information across CpGs and experimental conditions, which leads to better estimation of batch parameters and more stable adjustment, particularly when the number of samples in each batch is small. This is particularly useful to the Illumina Methylation BeadChip data, as we often see batch effects occurring at chip level, 12 samples a unit. Our data indicated that it not only successfully removed the remaining batch effects but also significantly increased biological signal detection. Even for the data with minor batch effects, it can further reduce technical variability on the top of normalization (Figure 1F). As it is an R function, it can be easily integrated with other packages such as "lumi" and other normalization procedures.

For an illustration, we applied the DWD approach to our Dataset 2 and 3. As expected, we found that it not only corrected batch effects but also removed certain biological signals. The numbers of differentially methylated CpGs associated with the study of interest were all lower compared to the EB corrected data regardless of normalization method used (Additional file 3). Similar to gene expression data, the DWD could change the scale of the methylation data, making the normalized data not directly comparable. It needed to be run two batches at a time and it would be cumbersome when multiple batches are involved.

It should be emphasized that a good study design is critical for evaluation and correction of batch effects. Samples from different study groups should be randomly or evenly allocated to different batches. If batches and study groups are totally confounded, i.e., one group of samples allocated on one batch and another group of samples on another, it would be impossible to separate batch from biological effects. Normalization might be the only option that can be exercised. With balanced design, batch factor and covariates such as sample group of study can be incorporated for more accurate adjustment.

The procedure of normalization and batch correction in this work is sequential, i.e., normalization is conducted first and batch correction is added subsequently. A comprehensive model that incorporates normalization, batch effect correction, and other important factors such as tissue heterogeneity might lead to a better result as proposed for gene expression microarray [32]. Although this evaluation was based on the Human Methylation27 platform, we expect these principles should be applicable to the new release of the 450 k Infinium Methylation Beadchip as both platforms use the similar Infinium methylation assay. To our experience, this bead based platform is sensitive to batch effects.

## Conclusion

In summary, we have demonstrated that genome-wide methylation data from Infinium Methylation BeadChip can be susceptible to various batch effects, which can profoundly affect downstream analyses and conclusions. Careful examination of such effects is necessary. All three commonly used normalization approaches reduce batch effects, but none of them can remove it completely, particularly when batch effects are substantial. QNβ is more aggressive yet effective when batch effects are severe; Lumi normalization performs better for the data with minor technical variation but worse for the data with obvious batch effects. ABnorm performed between the two. Batch effects affect CpG methylation values in an irregular way and specialized correction techniques are needed. EB correction along with normalization dramatically improves biological signal detection and is recommended for effective batch effect removal.

## Conflicts of interest

The authors declare that they have no competing interests.

## Authors' contributions

ZS and JPK conceived the study. ZS and HSC analyzed the data. TMT provided statistical support and guidance. YW, WMW, KVD, CJK and VDJ collected samples and generated the methylation data; ZS drafted the manuscript. All authors read, revised and approved the manuscript.

## Pre-publication history

The pre-publication history for this paper can be accessed here:

http://www.biomedcentral.com/1755-8794/4/84/prepub

## Supplementary Material

Additional file 1 **Fitted lowess curves of M-A plot for Dataset 2 and 3**. X-axis is for the methylation mean across all samples and Y-axis is the difference between each sample and the mean. Each curve represents a sample; red and green mark samples from two different batches. A: Dataset 2, red for Chip11 and green for Chip12. B: Dataset 3, red for Chip54 and green for Chip36. Bothe datasets show clear non-linear "intensity dependent" biases. Click here for file

Additional file 2 **Differential methylation p value distribution of positive and negative CpGs between prostate cancer and normal samples for Dataset 3 before and after normalization/batch correction**. The positive CpGs (85) were selected from genes frequently reported in the literature whose CpGs are hypermethylated in prostate cancer. The negative CpGs (358) were selected for housekeeping genes. A: After normalization and normalization/EB correction, the numbers of differentially methylated positive CpGs all increase compared to un-normalized data. B: The p values for negative CpGs are almost uniformly distributed and there is no indication of bias introduced from normalization and batch correction (the significant CpGs at p < 0.05 are all less than expected 18). Click here for file

Additional file 3 **Batch effect correction by distance weighted discrimination (DWD)**. DWD effectively removes batch effects. However, the numbers of significant CpGs associated with outcome of study are all lower than EB corrected data in Table 1 of the main text. Failure to incorporate biological covariates in the adjustment model is likely to compromise true biological signals. Click here for file
