# Supplementary figures and images for: Batch effect correction for genome-wide methylation data with Illumina Infinium platform
Source: BMC Med Genomics. 2011 Dec 16;4:84. doi: 10.1186/1755-8794-4-84 (PMC3265417; doi:10.1186/1755-8794-4-84)

**A**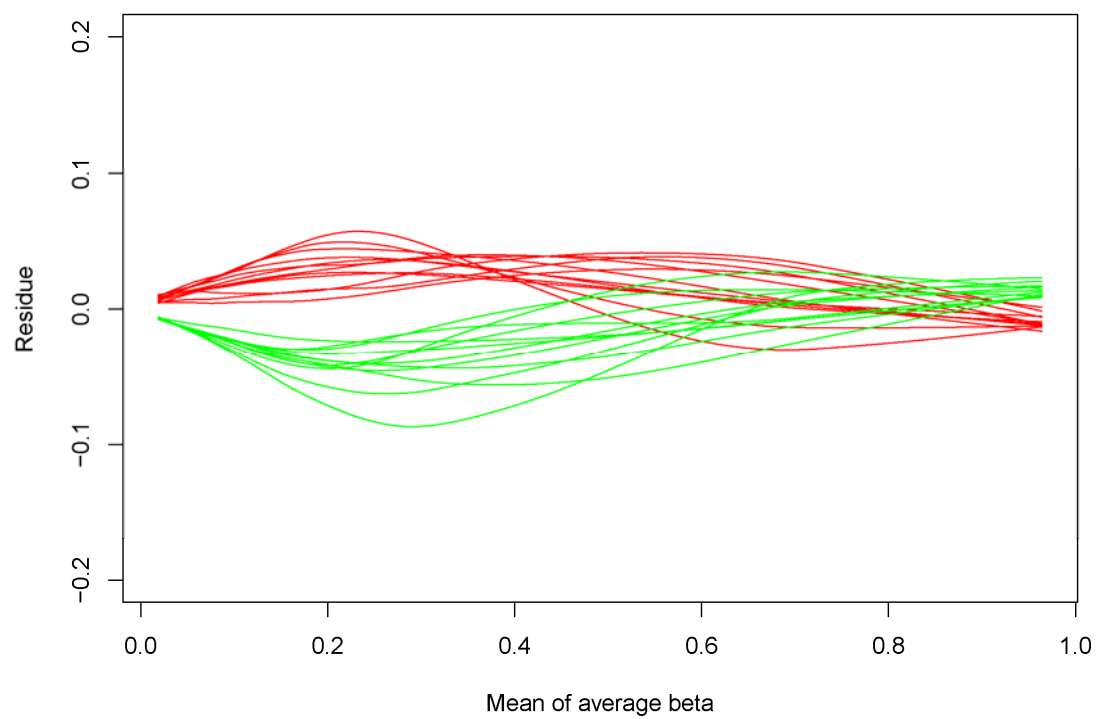**B**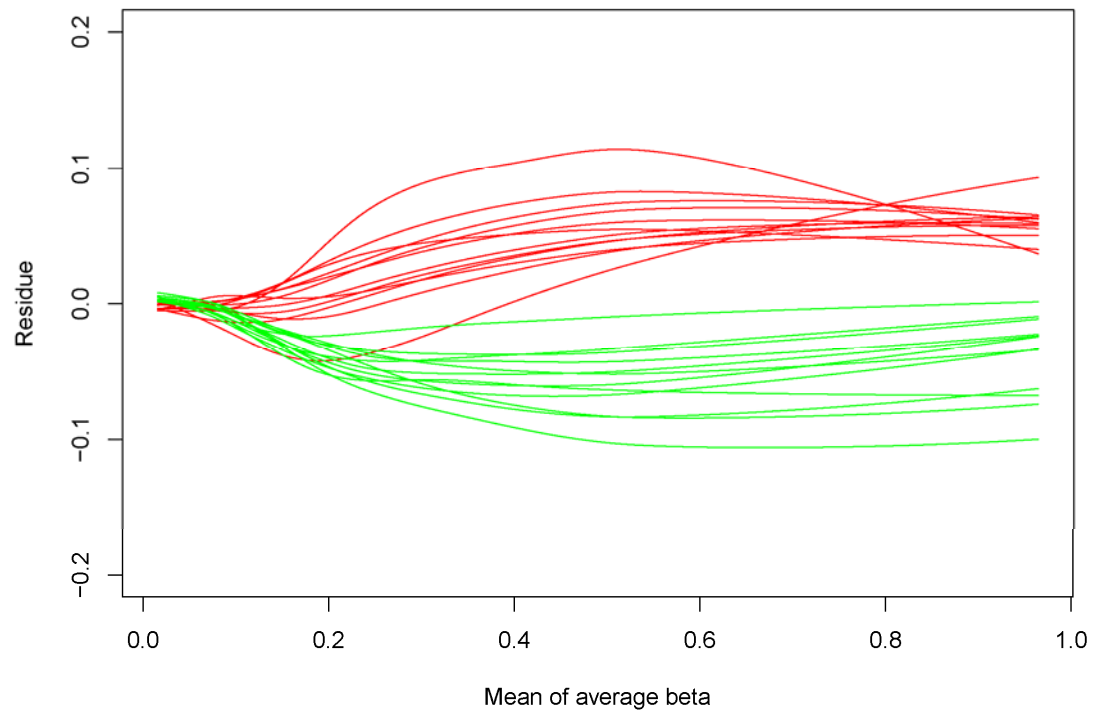

Supplement: Additional file 1 — Fitted lowess curves of M-A plot for Dataset 2 and 3. X-axis is for the methylation mean across all samples and Y-axis is the difference between each sample and the mean. Each curve represents a sample; red and green mark samples from two different batches. A: Dataset 2, red for Chip11 and green for Chip12. B: Dataset 3, red for Chip54 and green for Chip36. Bothe datasets show clear non-linear "intensity dependent" biases. [file 1755-8794-4-84-S1.PDF]

A: Positive Control CpGs

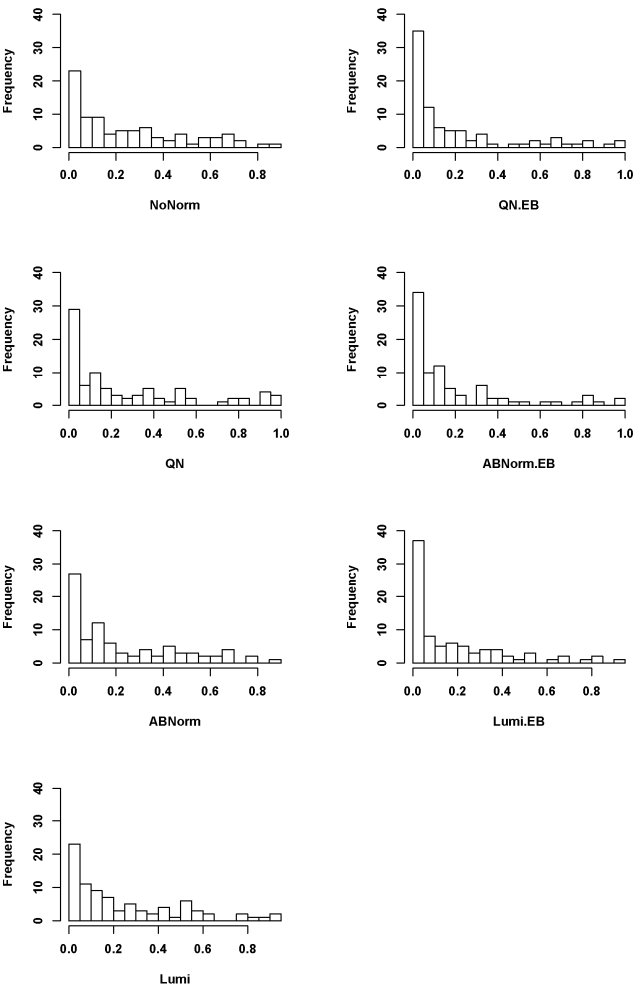

B: Negative Control CpGs

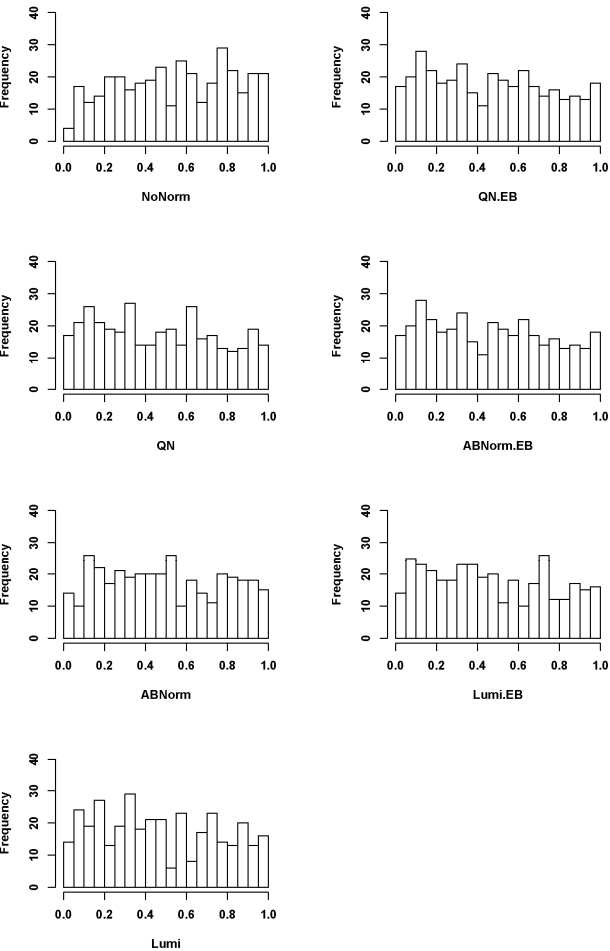

Supplement: Additional file 2 — Differential methylation p value distribution of positive and negative CpGs between prostate cancer and normal samples for Dataset 3 before and after normalization/batch correction. The positive CpGs (85) were selected from genes frequently reported in the literature whose CpGs are hypermethylated in prostate cancer. The negative CpGs (358) were selected for housekeeping genes. A: After normalization and normalization/EB correction, the numbers of differentially methylated positive CpGs all increase compared to un-normalized data. B: The p values for negative CpGs are almost uniformly distributed and there is no indication of bias introduced from normalization and batch correction (the significant CpGs at p < 0.05 are all less than expected 18). [file 1755-8794-4-84-S2.PDF]
