# Supplementary material for: Batch effect correction for genome-wide methylation data with Illumina Infinium platform
Source: BMC Med Genomics. 2011 Dec 16;4:84. doi: 10.1186/1755-8794-4-84 (PMC3265417; doi:10.1186/1755-8794-4-84)
Supplement: Additional file 3 — Batch effect correction by distance weighted discrimination (DWD). DWD effectively removes batch effects. However, the numbers of significant CpGs associated with outcome of study are all lower than EB corrected data in Table 1 of the main text. Failure to incorporate biological covariates in the adjustment model is likely to compromise true biological signals. [file 1755-8794-4-84-S3.PDF]

Dataset	Statistical measure	QNβ+ DWD	Lumi+ DWD	ABnorm+ DWD
**2**	Number (%) of CpGs associated with batch at p<0.01	0	0	0
	PCs associated with batch(% variance explained)*	None	None	None
	Number (%) of differentially methylated CpGs between case and control at p<0.01	892 (3.4)	955 (3.6)	884 (3.3
**3**	Number (%) of CpGs associated with batch at p<0.01	0	0	0
	PCs associated with batch (% variance explained)	None	None	None
	Number (%) of differentially methylated CpGs between cancer and normal at p<0.01	2,491 (9.4)	1,872 (7.1)	1,968 (7.4)

QNβ: quantile normalization at average β values; lumi: two step quantile normalization at probe signals implemented in R package "lumi"; ABnorm: quantile normalization for A and B signal separately; DWD: distance weighted discrimination. * The principal components (PC) significantly associated with batch effects at p value < 0.01 from the top 10 evaluated by Wilcoxon test and the percentage of variance the PC explains.
